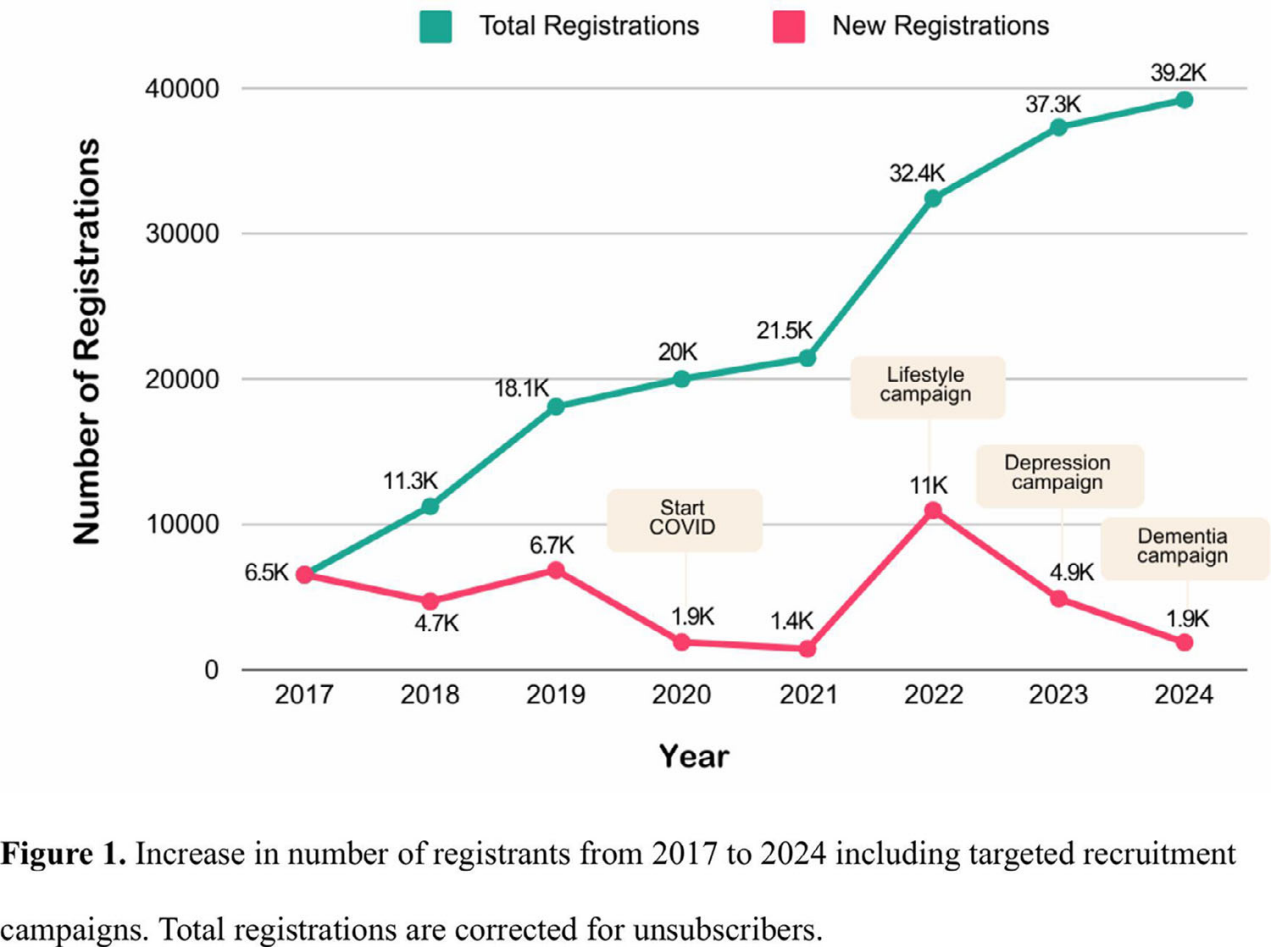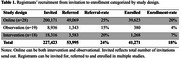# The Dutch Brain Research Registry’s progress and developments

**DOI:** 10.1002/alz70859_098470

**Published:** 2025-12-25

**Authors:** Marissa D. Zwan, Lisa Waterink, Florence I. Van der Zee, Tessa Jansen, Daphne Nijland, Flora H. Duits, Sietske A.M Sikkes, Wiesje M. van der Flier

**Affiliations:** ^1^ Amsterdam Neuroscience, Neurodegeneration, Amsterdam Netherlands; ^2^ Alzheimer Center Amsterdam, Neurology, Vrije Universiteit Amsterdam, Amsterdam UMC location VUmc, Amsterdam Netherlands; ^3^ Alzheimer Center Amsterdam, Neurology, Vrije Universiteit Amsterdam, Amsterdam UMC Location VUmc, Amsterdam, The Netherlands, Amsterdam Netherlands; ^4^ Amsterdam Neuroscience, Neurodegeneration, Amsterdam, The Netherlands, Amsterdam Netherlands; ^5^ Alzheimer Center Amsterdam, Department of Neurology, Amsterdam Neuroscience, Vrije Universiteit Amsterdam, Amsterdam UMC, Amsterdam Netherlands; ^6^ Faculty of Behavioural and Movement Sciences, Department of Clinical, Neuro and Developmental Psychology, Vrije Universiteit Amsterdam, Amsterdam Netherlands; ^7^ Epidemiology and Data Science, Vrije Universiteit Amsterdam, Amsterdam UMC location VUmc, Amsterdam Netherlands; ^8^ Alzheimer Center, Department of Neurology, Amsterdam UMC, Vrije Universiteit Amsterdam, Amsterdam Neuroscience, Amsterdam Netherlands; ^9^ Amsterdam Neuroscience, Vrije Universiteit Amsterdam, Amsterdam UMC, Amsterdam Netherlands

## Abstract

**Introduction:**

The Dutch Brain Research Registry (DBRR) is an online platform that supports researchers with recruitment and prescreening from the general population for brain‐related studies. The DBRR recruits individuals aged 18 and older with or without a brain disease. Our aim is to provide insight into recruitment and enrollment of participants for studies, registry growth, highlight recent platform developments, and discuss key challenges and successes.

**Methods:**

Registrants’ recruitment between November 2019 and December 2024 is presented in referral‐rate (number invited / number referred) and enrolment‐rate (number enrolled / number referred) for studies categorized in 1) online, 2) observational and 3) intervention. Targeted campaigns were conducted to increase number of registrants with prevention potential, depression and dementia diagnosis. Developments involved the launch of a study partner‐portal for patient support, and APOE‐genotyping to optimize prescreening and storage of biomaterials for future studies (DBRR Biobank).

**Results:**

As of 2017, the DBRR includes over 39,000 registrants (73%F, age 64±13 and 35% low educated). Since November 2019, participants have been recruited for 65 studies (28 online, 19 observational and 18 intervention), with a total of 53,995 referrals and 41,271 enrolments. Recruitment for online studies was most effective (Table 1). On average, registrants participated in 3.7±2.6 studies. Recruitment campaigns targeting healthy volunteers resulted in more registrations compared to patient groups (prevention potential *n*=13,795; depression *n*=978 and dementia *n*=375; Figure 1). In the first month post‐launch the partner‐portal, 362 signed up with a study partner. As part of the DBRR Biobank, 2,558 registrants have known APOE‐status and 1,854 provided material for the Biobank.

**Discussion:**

The DBRR was successful in recruiting participants for brain‐related studies in the Netherlands, as demonstrated by the significant growth of the registry and numerous studies facilitated. Its primary success lies in being able to facilitate a wide range of studies, including those focused on psychiatry and neurodegenerative conditions. Key challenges are ensuring the platform's long‐term sustainability, improving diversity among registrants, and effectively reaching specific patient groups.